# Glial Cells Promote Myelin Formation and Elimination

**DOI:** 10.3389/fcell.2021.661486

**Published:** 2021-05-11

**Authors:** Alexandria N. Hughes

**Affiliations:** Section of Developmental Biology, Department of Pediatrics, University of Colorado, Aurora, Aurora, CO, United States

**Keywords:** myelin, oligodendrocyte, glial-glial interactions, astrocyte, microglia

## Abstract

Building a functional nervous system requires the coordinated actions of many glial cells. In the vertebrate central nervous system (CNS), oligodendrocytes myelinate neuronal axons to increase conduction velocity and provide trophic support. Myelination can be modified by local signaling at the axon-myelin interface, potentially adapting sheaths to support the metabolic needs and physiology of individual neurons. However, neurons and oligodendrocytes are not wholly responsible for crafting the myelination patterns seen *in vivo*. Other cell types of the CNS, including microglia and astrocytes, modify myelination. In this review, I cover the contributions of non-neuronal, non-oligodendroglial cells to the formation, maintenance, and pruning of myelin sheaths. I address ways that these cell types interact with the oligodendrocyte lineage throughout development to modify myelination. Additionally, I discuss mechanisms by which these cells may indirectly tune myelination by regulating neuronal activity. Understanding how glial-glial interactions regulate myelination is essential for understanding how the brain functions as a whole and for developing strategies to repair myelin in disease.

## Introduction

Communication in the central nervous system (CNS) depends on faithful and timely action potential (AP) propagation along neuronal axons. These demands are in part met by myelin, a proteolipid-rich membrane that insulates axons to increase conduction velocity. Myelin is an evolutionary solution that reconciles the CNS need for speedy conduction with the size limitations of the animal brain: Insulation increases conduction velocity exponentially more effectively than increasing axon diameter, and takes only a fraction of the space. Given this advantage, it is perhaps unsurprising that myelin has evolved at least seven independent times among animals (Hartline and Colman, 2007). Nearly all of these myelin analogs share a similar structure and organization, including multilamellar membrane wraps that extend along axons, punctuated by gaps (nodes of Ranvier). With the exception of copepods, a remarkable feature of myelin membrane is that it is not produced by neurons (Wilson and Hartline, 2011). Instead, myelin is formed by glial cells. In the CNS of vertebrates, the myelinating glia are oligodendrocytes. The non-neuronal origin of a substance integral for neuronal AP propagation is consistent with the possibility that glia are conserved calibrators of neural circuits, optimizing circuit timing and function.

Myelin wraps axons to increase conduction velocity. Something underappreciated about myelination is variability: Oligodendrocytes can form different numbers of myelin sheaths, with different lengths and thicknesses. Consequently, axons display substantial variability in myelin patterning and coverage. Some axons are not myelinated, and among those that are, myelination can be complete or intermittent (Tomassy et al., 2014). Intriguingly, all of these varying parameters, including oligodendrocyte differentiation, myelin sheath number, length, and thickness, and axon selection can be promoted by neuronal activity, a subject that has been reviewed extensively (Fields, 2015; Bergles and Richardson, 2016; Hughes and Appel, 2016; Almeida and Lyons, 2017; Mount and Monje, 2017; Thornton and Hughes, 2020). But while neuronal activity can promote myelination, myelin variability persists in experiments in which activity is blocked (Etxeberria et al., 2016) or neurons are replaced with nanofibers (Bechler et al., 2015), indicating that activity is only one of potentially numerous factors that foster the tremendous variability of myelin. What other factors contribute to myelin variability? An emerging body of work indicates that other glial cells, including astrocytes, microglia, and cell types of the vasculature, are also critical myelin cultivators. By promoting oligodendrocyte differentiation, providing materials from which to build myelin, and pruning sheaths, glial cells are intimately involved in nurturing myelin throughout development.

Reductionist approaches have been essential for dissecting the individual roles of glia in the nervous system. These approaches have typically centered on neurons—for example, assaying neuronal physiology upon specific glial manipulations—because it is well accepted that altered neuronal function might change CNS function. Here, I evaluate the literature from a different reductionist standpoint. In this review, I center on myelinating oligodendrocytes and explore how other glial cells support the growth and elimination of myelin sheaths. I cover what is known about regulation of myelin sheaths by other glia, including astrocytes, microglia, and cell types of the vasculature. Specifically, I discuss the contributions of these cell types to myelin synthesis and remodeling of myelin. I take the liberty of combining findings that span model systems, CNS regions, and developmental stages and disease states (e.g., remyelination paradigms) with the goal of identifying common functional roles for glia that may transcend specific experimental constraints. Additionally, I discuss how these cell types may regulate neuronal activity to induce activity-dependent plastic changes in myelin. I begin by providing an overview of relevant phases of oligodendrocyte differentiation, myelination, and remodeling, and the following sections on glial interactions with myelin will follow this developmental organization.

## Oligodendrocyte Development and Plasticity

**FIGURE 1:**
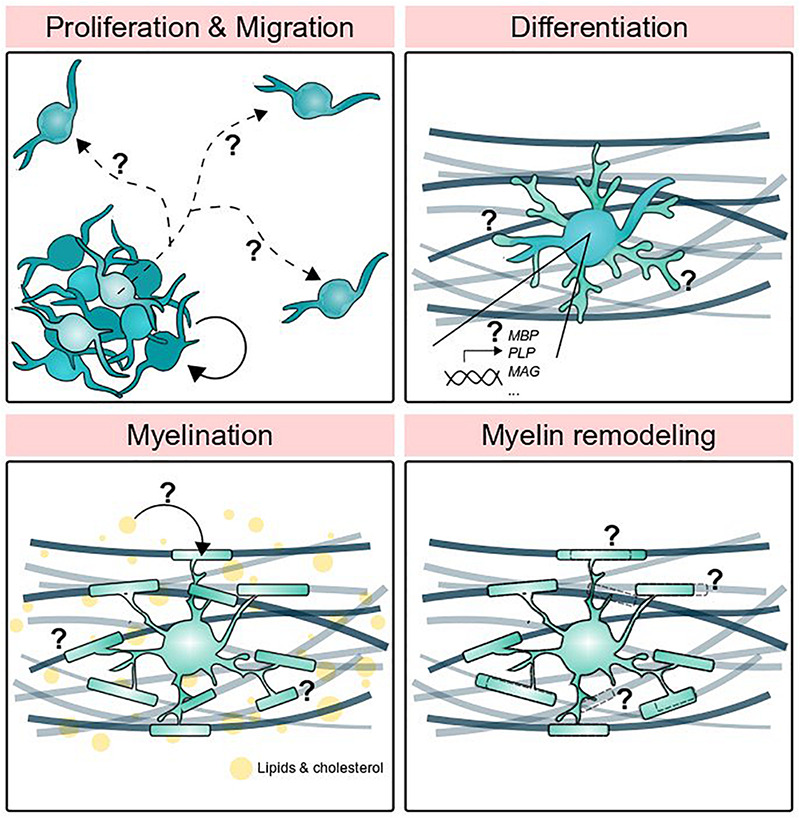
Stages of oligodendrocyte development supported by other glial cells. Oligodendrocyte precursor cells proliferate and migrate, but what determines precise migratory routes and proliferation rate? How do oligodendrocytes differentiate and turn on myelin gene expression? Circulating lipids are a major component of myelin, but where do those lipids come from? Finally, how do myelin sheaths grow, shrink, and disappear altogether?

Oligodendrocytes develop from oligodendrocyte precursor cells (OPCs). During development, OPCs are specified and migrate toward target axons before they differentiate into myelinating cells. In the mammalian forebrain, OPCs are born and migrate from three germinative zones in successive waves (Kessaris et al., 2006), whereas in the spinal cord OPCs are born and migrate in two waves. In both of these regions, the first wave is located ventrally: the medial ganglionic eminence (MGE) in forebrain and the progenitor of motor neuron (pMN) domain of the ventral spinal cord. Later, the ventricular zone of the lateral ganglionic eminence (LGE) and cortex give rise to OPCs, and a dorsal population of spinal cord OPCs are born (Kessaris et al., 2006; Winkler et al., 2018). From these birth locations, OPCs migrate to evenly distribute in the CNS (Noll and Miller, 1993; Ono et al., 1995; Kirby et al., 2006; Hughes et al., 2013). What cues direct OPCs to migrate long distances to attain their final positions in the CNS (Figure 1)? Axon- and meningeal-derived cues, including Tgfβ-1 (Choe et al., 2014) and Eph/Ephrin signaling (Prestoz et al., 2004), are one source of cues. Another, more local interaction occurs when OPCs interact with the vasculature. In our section on Vasculature, I examine the role of OPC-vasculature interactions in directed migration.

Following migration, OPCs begin to differentiate. Premyelinating oligodendrocytes elaborate processes that wrap axons and begin to synthesize myelin proteins and lipids that comprise the myelin sheath. Whether OPCs that do not differentiate represent an equipotent reserve pool or are a distinct subset with yet unknown functions is not entirely clear. OPC numbers are maintained by homeostatic proliferation and differentiation (Kirby et al., 2006; Hughes et al., 2013) and in the absence of axonal targets OPC proliferation and survival are moderately reduced, indicating that the population is responsive and matched to target availability (Almeida and Lyons, 2016). However, recent work indicates that multiple OPC subtypes exist and are separable by transcriptomics, intracellular calcium signaling, and membrane ion channels and receptors (Marques et al., 2016; Spitzer et al., 2019; Marisca et al., 2020), raising the possibility that both a differentiation reserve pool and other functional pools exist. For the purposes of this review, I will focus on those cells that do eventually myelinate and point the reader toward reviews covering OPCs in more detail (Mangin and Gallo, 2011; Bergles and Richardson, 2016). What controls the onset of differentiation? Several regulatory factors have been identified (Elbaz and Popko, 2019), and OPCs appear to divide a specific number of times prior to differentiation, consistent with an intrinsic timer mechanism (Temple and Raff, 1986; Raff, 2007). However, extracellular cues are also critical for differentiation (Wheeler and Fuss, 2016). For example, the OPC intrinsic timer depends on PDGF from astrocytes (Raff et al., 1988). Do other glial-derived factors support differentiation? Astrocytes, microglia, and endothelial cells, typically absent from culture, secrete cues that promote differentiation and can guide processes toward axons.

A major manifestation of differentiation is the production of myelin. Myelin is a specialized membrane that differs from the plasma membrane, particularly enriched in lipids, comprising 75% of the dry weight of myelin (Nave and Werner, 2014), and proteins that organize myelin structure and adhesion to the axon. Perhaps because there are fewer methods available to study lipids compared to proteins (Muro et al., 2014), lipid localization and trafficking in myelin is somewhat a technical blind spot in myelin biology. Instead, investigations have studied the genes and proteins that regulate lipid synthesis, with the caveat that synthesized lipids may localize differently than the enzymes that synthesize them. This approach revealed that cholesterol is required for the earliest stages of axon wrapping: wrapping is almost entirely blocked by global loss of function mutation of *hmgcs1*, which encodes the rate-limiting enzyme for cholesterol synthesis (Mathews et al., 2014). Do oligodendrocytes synthesize cholesterol and other lipids autonomously? Mice with oligodendrocyte-specific deletion of fatty acid synthase (*Fasn*) had hypomyelination in various CNS regions, but this could be partially rescued by increasing dietary lipids (Dimas et al., 2019). Additionally, in mice with oligodendrocyte-specific deletion of *Fdft1*, the gene encoding squalene synthase necessary for cholesterol synthesis, myelin still contained cholesterol, consistent with the possibility that oligodendrocytes obtain cholesterol from another source (Saher et al., 2005). Indeed, lipid analysis of purified myelin has revealed that oligodendrocytes incorporate circulating lipids to build myelin (Nave and Werner, 2014; Camargo et al., 2017), consistent with the possibility that proximal neurons and glia also provide and influence the lipids available for myelin construction.

The advancement of myelin around the axon and successful subversion of previous layers requires coordinated adhesion, both between layers of myelin as well as adhesion between the myelin and axon. The proteins myelin basic protein (MBP) and proteolipid protein (PLP), which together account for 68% of total myelin protein (Jahn et al., 2020) regulate myelin sheath compaction by adhering internal and external membrane leaflets, respectively. MBP is also required for actin disassembly during wrapping to promote membrane spreading (Zuchero et al., 2015). To promote adhesion to the axon, adhesion proteins must be corralled into the lateral edges that will form the paranodal loops that adhere to the axon (Rasband and Peles, 2016). The most well-studied adhesion protein at the paranodal interface is neurofascin-155 (NF155), a glial protein that binds axonal contactin-1 (Cntn1) and contactin-associated protein (Caspr) (Gollan et al., 2003). Additional adhesion molecules from the immunoglobulin superfamily, including Tag-1, Cadm1, and Cadm4, also coordinate axon-myelin adhesion along the juxtaparanode and internode (Poliak et al., 2003; Traka et al., 2003; Elazar et al., 2018; Hughes and Appel, 2019). Manipulation of these adhesion molecules disrupts myelin sheath number, length, targeting to axons, and lamellar organization (Djannatian et al., 2019; Elazar et al., 2019; Garcia and Zuchero, 2019; Hughes and Appel, 2019; Klingseisen et al., 2019). Modification of adhesion complexes, either autonomously by the axon or myelin, or potentially by other glial cells, represents one way that myelination can be changed.

After initial axon wrapping, myelination is not finalized is continuously subject to turnover and updates throughout life. Carbon dating experiments suggest that human myelin and oligodendrocytes are generated and integrated over the lifespan (Yeung et al., 2014). Further changes in myelin can be spurred by experience. Learning how to juggle and play the piano, activities that engage select populations of neurons, are associated with white matter additions in relevant brain areas (Scholz et al., 2009). This activity-dependent myelin growth, or “myelin plasticity,” includes adaptation of existing myelin and addition of new myelin sheaths by existing cells (Duncan et al., 2018; Bacmeister et al., 2020; Yang et al., 2021) as well as proliferation and subsequent differentiation of new oligodendrocytes (Barres and Raff, 1993; Gibson et al., 2014). At the cellular level, optogenetic or chemogenetic stimulation of neurons (Gibson et al., 2014; Mitew et al., 2018) or expression of neurotoxins to silence neurons (Hines et al., 2015; Mensch et al., 2015; Wake et al., 2015; Koudelka et al., 2016) demonstrate that myelin adapts to neuronal activity. Another side of myelin plasticity is myelin elimination. Although mechanisms of elimination are incompletely resolved, elimination is often referred to as “retraction” (Fields, 2015; Hines et al., 2015; Mensch et al., 2015; Baraban et al., 2018; Krasnow et al., 2018; Bacmeister et al., 2020), “contraction” (Yang et al., 2021), or “pruning” (Liu et al., 2013). Do activity-dependent additions or loss of myelin impact CNS function? Recent findings are consistent with this possibility. New oligodendrocytes and new myelin are required for motor learning and memory preservation (McKenzie et al., 2014; Pan et al., 2020; Steadman et al., 2020; Wang et al., 2020). These systems-level adaptations are presumably driven by activity-dependent oligodendrocyte differentiation and myelin growth and remodeling on behaviorally relevant neurons. However, all glial cell types are responsive to neuronal activity (Barres and Raff, 1993; Demerens et al., 1996; Poskanzer and Yuste, 2011; Li et al., 2012; Nagy et al., 2017; Liu et al., 2019; Stowell et al., 2019; Zuend et al., 2020; Nagai et al., 2021), raising the possibility that other glial cells may promote or solidify adaptive changes by regulating properties of myelin. Getting the whole picture will require learning not only how cell types interact with one another in isolation, but also how these interactions moderate each other in the context of the whole brain.

## Astrocyte Interactions With the Oligodendrocyte Lineage and Myelin

**FIGURE 2:**
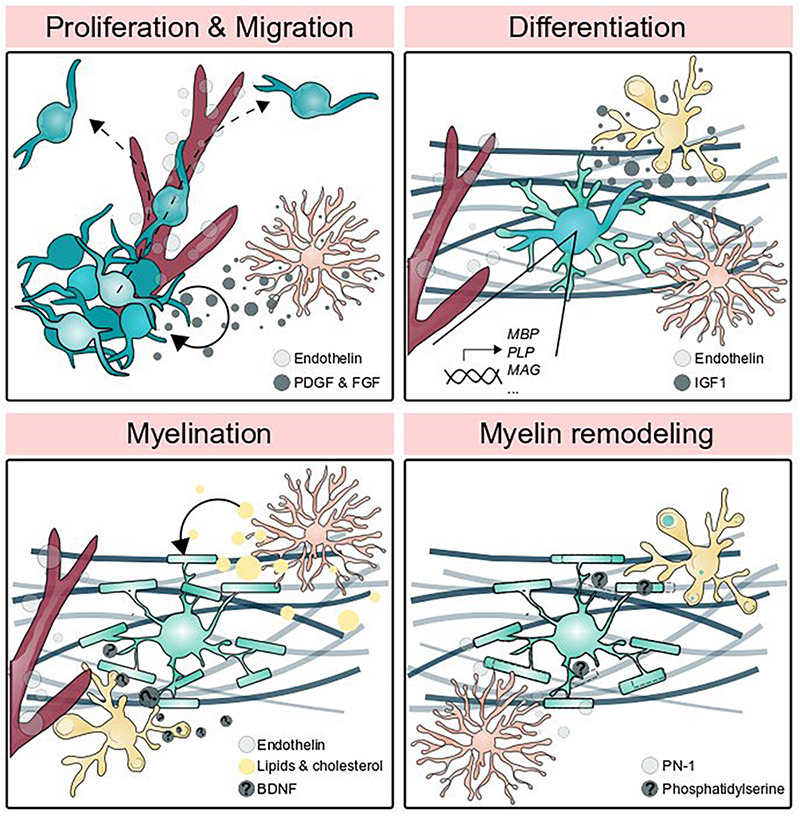
Glial cells promote myelin formation and elimination. Oligodendrocyte precursor cell proliferation is promoted by astrocytic PDGF and FGF signaling and OPCs migrate along the vasculature. Endothelin secreted by endothelial cells and IGF1 secreted by microglia promote differentiation, and astrocytes guide OPCs to axons. The generation of myelin membrane (which may be thought of as one feature of differentiation) utilizes lipids and cholesterol produced by astrocytes, BDNF secreted by neurons and potentially augmented by microglia-secreted BDNF, and endothelin signaling to the receptor EDNRB located on oligodendrocytes. Astrocytic secretion of PN-1 promotes myelin stability by inhibiting thrombin-mediated paranodal lifting, whereas microglia phagocytose myelin by detecting phosphatidylserine.

**FIGURE 3:**
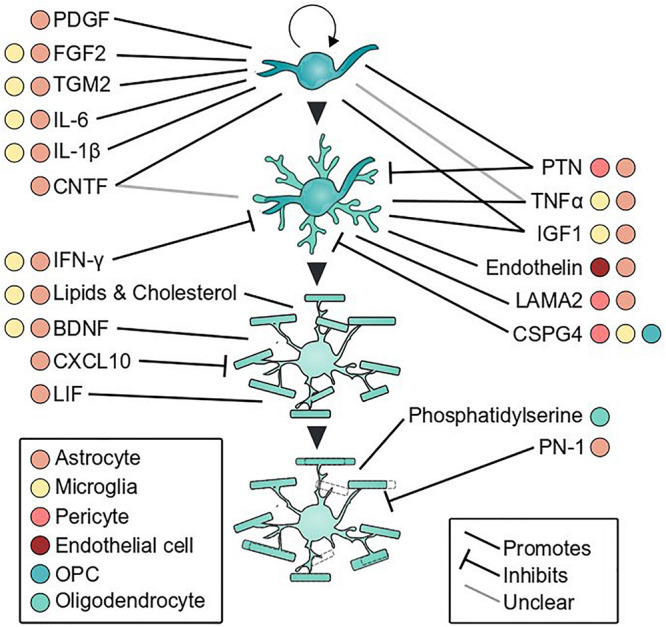
Glial secreted factors that influence oligodendrocyte development. Oligodendrocyte proliferation, differentiation, myelination, and myelin remodeling are shaped by cues secreted by glial cells. Colored dots indicate cell types that secrete each factor and line type denotes direction of effect. References for each factor are listed in Table 1.

Astrocytes develop before oligodendrocytes, both *in vivo* and in culture (Qian et al., 2000), consistent with the possibility that astrocytes influence the entire course of oligodendrocyte development. Astrocytes secrete PDGF and FGF, which promote the proliferation and impede the differentiation of OPCs (Barnett and Linington, 2013; Lundgaard et al., 2014; Figure 2). The relationship between OPCs and astrocytes during OPC differentiation is less well resolved. Several lines of evidence raise the possibility that astrocytes help control the timing of myelination. One mechanism that occurs during OPC differentiation is the translocation of hundreds of mRNAs into premyelinating processes, presumably to enable local translation of proteins relevant for sheath maturation (Thakurela et al., 2016). The archetypal mRNA studied in this process is *Mbp* mRNA, which translocates into myelinating processes both in culture and *in vivo* (Ainger et al., 1993; Herbert et al., 2017; Yergert et al., 2021). MBP is classically known for driving myelin compaction (Readhead et al., 1987) and has recently been implicated in actin disassembly during myelin wrapping (Zuchero et al., 2015), raising the possibility that locally translated MBP is integral to wrapping. *In vitro* labeling of *Mbp* mRNA in oligodendrocyte-astrocyte cocultures revealed that direct contact with astrocytes inhibited mRNA translocation into oligodendrocyte processes (Amur-Umarjee et al., 1993), but the presence of neurons alleviated this inhibition via PDGF-AB and -BB secretion (Amur-Umarjee et al., 1997). This is consistent with a role for astrocytic contact in limiting myelinating potential of processes when neurons are unavailable as substrates for myelination. Complementing this interpretation, co-cultured astrocytes guided oligodendrocyte processes to align with retinal ganglion cell axons (Meyer-Franke et al., 1999), increased the fraction of axons selected for myelination (Sorensen et al., 2008), and enhanced myelin growth and thickness (Watkins et al., 2008). Taken together, these studies raise the intriguing possibility that astrocytic contact may regulate the timing of myelination, ensuring that oligodendrocyte processes are aligned with axons before permitting mRNA translocation and presumably local synthesis of myelin proteins. Such a timing mechanism is also supported by astrocytic secretion of factors that both promote and inhibit myelin wrapping (Table 1 and Figure 3).

**TABLE 1:** Glial secreted factors that shape oligodendrocyte development.

**Factor**	**Glial cell types that produce factor**	**Oligodendrocyte developmental stage**	**Effect**	**References**
Platelet-derived growth factor (PDGF)	Astrocytes	Proliferation	Promotes	Noble et al., 1988; Raff et al., 1988; Richardson et al., 1988; Hart et al., 1989; Barnett and Linington, 2013; Lundgaard et al., 2014
Fibroblast growth factor-2 (FGF2)	Astrocytes, microglia, OPCs	Proliferation	Promotes	Liu et al., 1998; Messersmith et al., 2000; Fortin et al., 2005; Barnett and Linington, 2013; Kirby et al., 2013; Birey et al., 2015
Transglutaminase-2 (Tgm2)	Microglia, astrocytes	Proliferation	Promotes	Giera et al., 2018; Espitia Pinzon et al., 2019
Interleukin 6 (IL-6)	Microglia, astrocytes	Proliferation	Promotes	Barres et al., 1993; Lau and Yu, 2001; Taylor et al., 2010; Shigemoto-Mogami et al., 2014
Interleukin 1β (IL-1β)	Microglia, astrocytes	Proliferation	Promotes	Didier et al., 2004; Shigemoto-Mogami et al., 2014
Ciliary neurotrophic factor (CNTF)	Astrocytes	Survival, proliferation, differentiation (?)	Reports of both pro- and absent effect on differentiation	Barres and Raff, 1993; Barres et al., 1996; Stankoff et al., 2002; Albrecht et al., 2003; Nash et al., 2011
Pleiotrophin (Ptn)	Astrocytes, pericytes	Proliferation, differentiation	Promotes proliferation, suppresses differentiation	Yeh et al., 1998; Kuboyama et al., 2012, 2015, 2016; Mcclain et al., 2012; Nikolakopoulou et al., 2019
Insulin-like growth factor-1 (IGF1)	Microglia, astrocytes	Proliferation, differentiation	Promotes	Ye et al., 2002; Shigemoto-Mogami et al., 2014; Pitt et al., 2017; Wlodarczyk et al., 2017; Chen et al., 2019
Tumor necrosis factor-α (TNFα)	Microglia, astrocytes	Proliferation (?), differentiation	Reports of both pro- and anti-proliferative effects	Arnett et al., 2001; Lau and Yu, 2001; Nakazawa et al., 2006; Taylor et al., 2010; Su et al., 2011; Shigemoto-Mogami et al., 2014
Interferon γ (IFN-γ)	Microglia, astrocytes	Differentiation	Inhibits differentiation, induces cell stress and death	Baerwald and Popko, 1998; Laferla et al., 2000; Lau and Yu, 2001; Chew et al., 2005; Lin et al., 2006; Shigemoto-Mogami et al., 2014
Endothelin 1 (ET-1)	Endothelial cells, radial glia, astrocytes	Differentiation	Promotes	Ostrow et al., 2011; Swire et al., 2019; Adams et al., 2020
Laminin, α2 (LAMA2)	Pericytes, astrocytes?	Differentiation	Promotes	De La Fuente et al., 2017; Silva et al., 2019
Chondroitin sulfate proteoglycan 4 (CSPG4, NG2)	OPCs, pericytes, microglia	Differentiation	Favors differentiation of astrocytes at expense of oligodendrocytes	Baror et al., 2019; Huang et al., 2020; Liu et al., 2021
Lipids and Cholesterol	Astrocytes, microglia	Myelination	Promotes; microglia sterols support remyelination	Saher et al., 2005; Mathews et al., 2014; Camargo et al., 2017; Dimas et al., 2019; Werkman et al., 2020; Berghoff et al., 2021
Brain-derived neurotrophic factor (BDNF)	Astrocytes, microglia	Myelination	Promotes	Nakajima et al., 2002; Coull et al., 2005; Gomes et al., 2013; Parkhurst et al., 2013; Fulmer et al., 2014
Leukemia inhibitory factor (LIF)	Astrocytes	Myelination	Promotes	Barres et al., 1993; Butzkueven et al., 2002; Ishibashi et al., 2006; Moidunny et al., 2012
C-X-C motif chemokine ligand 10 (CXCL10)	Astrocytes	Myelination	Inhibits axon wrapping	Nash et al., 2011
Phosphatidylserine (PS)	Oligodendrocytes	Myelin remodeling	Eat-me signal for microglia	Djannatian et al., 2021
Protease nexin-1 (PN-1, SERPINE2)	Astrocytes	Myelin remodeling	Inhibits myelin lifting	Dutta et al., 2018

At myelination onset, astrocytes play a critical role in producing lipids for myelin synthesis. Recent evidence showed that astrocytes provide the majority of required lipids to oligodendrocytes (Camargo et al., 2017). Astrocyte-specific deletion of the major lipid biosynthesis regulator SREBP cleavage activating protein (SCAP) caused more severe and persistent hypomyelination than oligodendrocyte-specific loss of SCAP, which resolved after a brief developmental delay (Camargo et al., 2017). This is consistent with previous findings from a mouse model of oligodendrocyte-specific deletion of *Fdft1*, the gene encoding squalene synthase, also required for cholesterol synthesis, which exhibited early hypomyelination that also caught up by adulthood (Saher et al., 2005). Therefore, in addition to uptake of lipids and cholesterol from the extracellular environment (Saher et al., 2005; Nave and Werner, 2014; Camargo et al., 2017), oligodendrocytes build myelin primarily from astrocyte-derived lipids. The transfer mechanism is incompletely resolved and could involve direct transfer or uptake of astrocyte-derived lipids and cholesterol from the extracellular milieu. Intriguingly, lipid availability may vary with regional astrocyte heterogeneity. Gray matter astrocytes may secrete more cholesterol than white matter astrocytes, and inhibiting cholesterol synthesis from white matter-derived astrocytes improved *in vitro* myelination (Werkman et al., 2020), raising the possibility that different populations of astrocytes provide different support for nearby oligodendrocytes. Together, these data extend the known role of astrocytes in providing cholesterol to neurons to promote synapse formation (Mauch et al., 2001) to include oligodendrocytes to enable myelin formation.

Oligodendrocytes and astrocytes maintain contact through somatic and lamellar gap junctions, which might promote myelination and serve to ensure ionic balance. These heterotypic gap junctions can be formed by astrocytic expression of Cx30 or Cx43 and oligodendroglial expression of Cx32 or Cx47 (Nagy et al., 2003; Orthmann-Murphy et al., 2007b). Deletion of different subsets of these connexins has provided evidence that converges on a role for gap junctions in myelin formation or maintenance: loss of Cx30 and Cx43 was associated with myelin vacuolization (Lutz et al., 2009), recessive mutations in the gene encoding Cx47 cause Pelizaeus-Merzbacher-like disease, a dysmyelinating disease in humans (Orthmann-Murphy et al., 2007a, b), and loss of Cx32 was associated with cortical myelin defects and hyperexcitability (Sutor et al., 2000). Mechanistically, a precise role for O-A coupling in myelination has been elusive. Deletion of the genes encoding Cx32 and Cx47 in oligodendrocytes was associated with dysregulation of lipid synthesis genes and an immune response (Wasseff and Scherer, 2015) and loss of Cx30 and Cx43 reduced levels of Mbp in corpus callosum (Lutz et al., 2009), raising the possibility that O-A coupling supports myelin gene expression. A potential confound to this interpretation is that loss of coupling may leave oligodendrocytes susceptible to excitotoxic damage, and myelin reductions observed in these models might reflect injury rather than impaired development. Indeed, O-A junctions allow K^+^ influx in oligodendrocytes and astrocytes to diffuse into the syncytium, protecting coupled cells from high concentrations of K^+^ and excitotoxic damage (Nagy and Rash, 2000; Menichella et al., 2006; Battefeld et al., 2016). Distinguishing the relative contributions of gap junction coupling to promoting myelination and protecting cells from excitotoxic damage has the potential to teach us a lot about oligodendrocyte development and heterogeneity, especially given the vast regional variation in O-A coupling frequency (Wasseff and Scherer, 2011).

Astrocytes are also emerging as regulators of myelin remodeling. Over 95% of nodes of Ranvier are contacted by astrocytes (Serwanski et al., 2017), a position that grants astrocytes proximity to many neighboring myelin sheaths. Exocytosis of a thrombin inhibitor, protease nexin-1 (PN-1, encoded by *Serpine2*) from astrocytes prevented thrombin-mediated cleavage of NF155 (Dutta et al., 2018), which anchors myelin paranodal loops to the axon. Thrombin is expressed by neurons and can also enter the CNS from the vasculature. At steady state, some NF155 was cleaved, evidenced by myelin lifting at 20% of observed paranodes. Blocking astrocytic exocytosis to prevent PN-1 secretion further increased the number of detached paranodes, consistent with the possibility that PN-1 promotes sheath stability by preventing thrombin-mediated NF155 cleavage. Under the model proposed by the authors, thrombin can increase paranodal lifting to allow for resorption of the outermost layer of myelin by the oligodendrocyte cell body. The resorption of myelin should be distinguished from other ways that astrocytes have been shown to remodel myelin, such as via phagocytosis. Astrocyte-like radial glia phagocytose optic nerve myelin in frogs (Mills et al., 2015), raising the possibility that both paranodal lifting and sheath phagocytosis contribute to sheath remodeling. Further investigation of these mechanisms *in vivo* might better resolve the conditions that contribute to different mechanisms of remodeling, potentially including regional astrocyte availability and heterogeneity (Bayraktar et al., 2015).

## Microglial Interactions With Oligodendrocyte Lineage Cells and Myelin

Microglia are resident immune cells of the CNS that differentiate from macrophages. Tools that allow manipulation of macrophages and microglia have been invaluable for uncovering how microglia contribute to oligodendrocyte development and myelination. Microglia development is covered in more depth elsewhere (Nayak et al., 2014), but here I introduce features that are relevant to manipulation. Erythromyeloid progenitors in the yolk sac are specified to become either macrophages or neutrophils in an interferon regulatory factor 8 (IRF8) -dependent manner (Holtschke et al., 1996; Scheller et al., 1999; Shiau et al., 2015). A subset of yolk sac macrophages will then invade the CNS and differentiate into microglia. The distribution and survival of microglia within the CNS depends on the function of the receptor CSF1R, which binds ligands IL-34 and CSF-1 (Ginhoux et al., 2010; Erblich et al., 2011; Oosterhof et al., 2018). Whereas both IRF8 and CSF1R disruption have allowed investigators to study how the CNS develops without microglia, CSF1R inhibition is increasingly popular due to the development of inhibitors for this receptor (Elmore et al., 2014). However, CSF1R manipulation also affects peripheral macrophages (Lei et al., 2020; Green and Hume, 2021), and the search for more specific tools has continued. By deleting a super-enhancer in the CSF1R locus, Rojo et al. (2019) generated a new mouse model that lacks brain microglia and a few other populations of macrophages but CSF1R^ΔFIRE/ΔFIRE^ mice are healthy and fertile. Additionally, a transmembrane protein, TMEM119, is expressed in microglia but not macrophages (Bennett et al., 2016; Satoh et al., 2016) and recently TMEM119 mouse lines have been generated to label and manipulate microglia (Kaiser and Feng, 2019). However, TMEM119 is not expressed by microglia in all model organisms and is notably absent from zebrafish and chicken microglia (Geirsdottir et al., 2019), its expression decreases in inflammatory conditions (Bennett et al., 2016), and it is expressed by a subset of peripheral macrophages during development (Grassivaro et al., 2020). P2RY12 is another promising marker that appears restricted to microglia and dural and choroid plexus macrophages, but not other macrophages (McKinsey et al., 2020). Although all of these existing methods of targeting microglia have limitations, it was recently revealed that microglia-fated macrophages are transcriptionally distinguishable within the yolk sac (Utz et al., 2020). Newly identified markers for yolk sac microglia-fated macrophages may enable the discovery of new targets for earlier and more specific perturbation of the lineage (Utz et al., 2020).

Microglia secrete cues that promote OPC proliferation, differentiation, and myelination. By ablating microglia during early postnatal development (P2-P7) with the CSF1R inhibitor BLZ945, Hagemeyer et al. (2017) found that microglia maintain OPC numbers and myelination in corpus callosum and cerebellum (Hagemeyer et al., 2017). Mice without microglia had fewer OPCs during development and by early adulthood had reduced myelin. Some of the phenotypes varied between brain regions, which could reflect regional heterogeneity in oligodendrocytes (Marques et al., 2016) or microglia (De Biase et al., 2017; Hammond et al., 2019; Li et al., 2019). Indeed, a specific subset of microglia that express *Cd11c* are present in developing white matter and ablation or conditional knockout of *Igf1* in these cells also impaired myelination (Wlodarczyk et al., 2017). Together, these data suggest that a subset of microglia promote myelination through IGF1 production. Other signaling pathways between microglia and OPCs may also modify myelin development. Giera et al. (2018) found that an adhesion G-protein coupled receptor, GPR56, located on OPCs, interacts with Transglutaminase-2 (TGM2) secreted by microglia to promote OPC proliferation in the presence of the extracellular matrix (ECM) protein laminin-111 (Giera et al., 2018). Importantly, remyelination following a demyelinating lesion was impaired in knockout mice lacking TGM2—GPR56 signaling, suggesting that this microglia-OPC-ECM signaling axis is essential not only for OPC numbers but functional remyelination (Giera et al., 2018). Astrocytes also express TGM2, raising the possibility that regions with differential densities of astrocytes and microglia maintain signaling via this signaling axis (Espitia Pinzon et al., 2019). Furthermore, microglial deposition of the ECM molecule CSPG4 in aging shifts the microenvironment to favor the differentiation of NG2 cells into astrocytes at the expense of oligodendrocytes (Baror et al., 2019). Taken together, microglia-ECM interactions may promote OPC proliferation and differentiation in development but increasingly inhibit OPC differentiation in later life.

In addition to IGF1 production, microglia are secretory cells that generate a variety of molecules and cytokines that have been studied in regulation of neuronal and synaptic activity (York et al., 2018). However, some of these secreted molecules have been independently implicated in regulating myelination. For example, microglia secrete BDNF (Nakajima et al., 2002; Coull et al., 2005; Gomes et al., 2013; Parkhurst et al., 2013), which has been found to promote myelination in numerous contexts (Mctigue et al., 1998; Lundgaard et al., 2013; Geraghty et al., 2019). The contribution of microglia-derived BDNF to myelination has not been investigated, but astrocyte-derived BDNF supports remyelination after cuprizone-mediated demyelination (Fulmer et al., 2014), consistent with the possibility that microglial-derived BDNF also promotes myelination. Microglia also secrete factors including TNFα, IL-6, FGF2, IL-1β, and IFN-γ that stimulate OPC proliferation and differentiation (Shigemoto-Mogami et al., 2014; Miron, 2017), but precise roles for these secreted cues in myelination have yet to be investigated.

Recent investigations of microglial heterogeneity have identified a population of white matter-associated microglia present during early postnatal development in mouse (Hammond et al., 2019; Li et al., 2019; McNamara and Miron, 2020). The role of this microglia subset is not yet known, but microglia in this subset (*Clec7a*+) contain *Mbp* transcripts (Li et al., 2019), raising the possibility that white matter microglia phagocytose myelin or oligodendrocytes. Microglia phagocytose myelin in disease models and in culture (Trotter et al., 1986; van der Laan et al., 1996; Smith and Hoerner, 2000), but a role for myelin engulfment during normal development is only beginning to be understood. We recently used zebrafish to investigate myelin phagocytosis by microglia during development (Hughes and Appel, 2020). We found that microglia dynamically engage with sheaths in myelinated tracts and phagocytose a subset of nascent sheaths. This may imply selective expression of a cue. Intriguingly, a recent preprint identified phosphatidylserine (PS), a known eat-me cue at synapses (Li et al., 2020; Park et al., 2020; Scott-Hewitt et al., 2020), as a likely cue for developmental myelin phagocytosis (Djannatian et al., 2021). Similar to synapse elimination, the cues that direct myelin phagocytosis may vary between brain regions (Gunner et al., 2019). Furthermore, emerging non-phagocytic microglial mechanisms of synapse modification, such as local extracellular matrix modification, may also impact myelin growth and loss (Cheadle et al., 2020; Nguyen et al., 2020). Identifying the cues that regulate myelin phagocytosis and non-phagocytic elimination, and the possibility that those cues may overlap or be distinct from cues regulating synapse elimination has the possibility to teach us a great deal about general principles of brain wiring during development.

## Vascular Interactions With the Oligodendrocyte Lineage and Myelin

In addition to astrocytes and microglia, cell types of the vasculature also interact with myelinating oligodendrocytes. The vasculature comprises two interacting cell types, endothelial cells and pericytes. During development, OPCs use the vasculature as a physical substrate for migration (Tsai et al., 2016) and crawl along it to distribute throughout the CNS. To learn which vascular cell type interacts with OPCs to promote migration, Tsai et al. (2016) used genetic approaches to ablate both endothelial cells and pericytes (*Gpr124*^–/–^), or pericytes only (*Pdgfrb*^–/–^) and studied OPC migration. They found that endothelial cells, and specifically endothelial expression of the adhesion G-protein coupled receptor, GPR124, were required for OPCs to migrate and distribute throughout the CNS. Within OPCs, CXCR4 and autocrine signaling of the Wnt ligands Wnt7a and -7b first promoted attraction to the vasculature, and were downregulated later, presumably allowing for detachment and differentiation (Yuen et al., 2014; Tsai et al., 2016).

Other factors produced by vascular cells may actively promote OPC differentiation and myelination. Endothelial cells produce endothelin, which was shown to promote oligodendrocyte differentiation in the subventricular zone (SVZ) (Adams et al., 2020). In a demyelination paradigm, pericyte secretion of the extracellular matrix protein LAMA2 could stimulate OPC differentiation (De La Fuente et al., 2017), raising the possibility that pericytes also promote differentiation during development via matrix deposition. Taken together, these data suggest a model by which OPCs migrating along vasculature both downregulate CXCR4 and Wnt ligands and are exposed to endothelin and possibly ECM factors to promote differentiation and myelination. In support of this model, Swire et al. (2019) found that upon oligodendrocyte-specific knockout of EDNRB, the endothelin receptor, oligodendrocytes formed fewer myelin sheaths (Swire et al., 2019). These authors also discovered that social isolation reduced myelination in prefrontal cortex, replicating a previously published result (Makinodan et al., 2012), and additionally found that social isolation reduced expression of endothelin by endothelial cells. An intranasal endothelin receptor agonist was sufficient to rescue the social deprivation-associated myelination defect, consistent with the possibility that activity-dependent myelination requires activation of EDNRB receptors on oligodendrocytes. Intriguingly, mice with oligodendrocyte-specific loss of EDNRB were less sociable than wildtype siblings, raising the possibility that endothelin signaling-dependent myelination promotes social behavior (Swire et al., 2019).

## On the Horizon: Glial Regulation of and by Neuronal Activity Shapes Downstream Activity-Dependent Myelination

**FIGURE 4:**
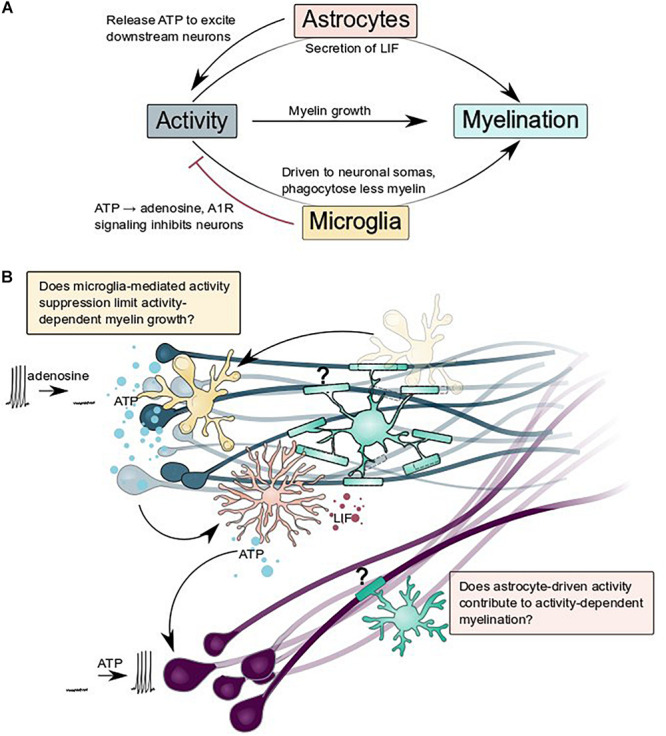
Astrocytes and microglia regulate and are regulated by neuronal activity: what are the consequences for activity-dependent myelination? **(A)** Summary schematic of interactions between astrocytes, microglia, neurons, and myelination described by Ishibashi et al. (2006), Badimon et al. (2020), Ma et al. (2016), and Hughes and Appel (2020). **(B)** Does glial regulation of neuronal activity change activity-dependent myelination? Does microglial suppression of activity (blue neurons) and astrocytic release of ATP to excite nearby neurons (purple) contribute to activity-dependent myelination?

Each glial cell type that I have discussed thus far regulates oligodendrocyte development, but are additionally regulated by and can regulate neuronal activity. How does modulation by and of neuronal activity impact myelination? Suppression and stimulation of activity in sparse axons changes the growth of myelin on those axons, ostensibly in a sheath- or oligodendrocyte-autonomous manner (Hines et al., 2015; Mensch et al., 2015; Wake et al., 2015; Koudelka et al., 2016; Mitew et al., 2018). How, then, does glial manipulation of axonal activity modulate the potential for activity-dependent myelination? Additionally, how does neuronal signaling to glia impact glial interactions with myelin (Figure 4)? These questions have been difficult to investigate because they require the inclusion and manipulation of multiple cell types and additional controls to isolate interactions of interest. Despite these challenges, such holistic investigations have begun to teach us how cell-cell interactions that have been identified by reductionist approaches interact with and moderate each other in the context of the whole brain (Liddelow et al., 2017; Geraghty et al., 2019; Gibson et al., 2019; Forbes et al., 2020). I conclude this review by raising questions that integrative approaches are now poised to tackle.

Glial cells can manipulate neuronal activity, both chronically and acutely. By participating in developmental synapse formation and refinement, glia broadly limit the range of activity that is possible within a nervous system. Emerging evidence suggests that glia can also act on much shorter timescales to flexibly modify activity. Astrocytes can increase activity, coupling the activity of populations of non-synaptically connected neurons via ATP secretion or other processes that occur downstream of intracellular calcium elevations (Ma et al., 2016; Mu et al., 2019). Might such synchronization of activity between disconnected neuronal populations configure similar activity-dependent myelination? Additionally, microglia can suppress neuronal activity, presumably via ATP hydrolysis and A1R signaling at somatic contacts on neurons (Li et al., 2012; Eyo et al., 2014; Badimon et al., 2020; Cserép et al., 2020). Does this suppression limit activity-dependent myelin growth in brain regions where microglia are particularly enriched? Finally, an emerging body of work implicates oligodendrocytes, astrocytes, and the vasculature in providing metabolic support for axons (Nave, 2010; Fünfschilling et al., 2012; Lee et al., 2012; Saab et al., 2016; Nortley and Attwell, 2017; Meyer et al., 2018; Philippot et al., 2021; Philips et al., 2021), enabling sustained neuronal activity and function. Intriguingly, fuel sources and types vary between brain regions (Meyer et al., 2018) and astrocytes might be particularly important for providing trophic support during development (Philips et al., 2021). In addition to glial control of neuronal activity, glial cells are also altered by activity and this may change how they interact with myelin. For example, astrocytes responded to ATP released by active neurons by secreting leukemia inhibitory factor (LIF) which could promote myelination (Ishibashi et al., 2006). In our recent paper, we found that microglia contacted active neuronal somas more frequently and phagocytosed less myelin from axons (Hughes and Appel, 2020). Together, the interplay of individual glial cell types with neurons and with each other may tune glial function and neuronal activity to optimize neural circuit function.

## Author Contributions

ANH conceptualized the review and wrote and illustrated the manuscript.

## Conflict of Interest

The author declares that the research was conducted in the absence of any commercial or financial relationships that could be construed as a potential conflict of interest.
